# Validation of a Virtual Environment to Induce State Social Physique Anxiety in Women with Obesity and Social Physique Anxiety

**DOI:** 10.3390/jcm12186065

**Published:** 2023-09-20

**Authors:** Alice Jeanningros, Aurélie Baillot, Giulia Corno, Marie-Christine Rivard, Annie Aimé, Stéphane Bouchard

**Affiliations:** 1 Psychoeducation and Psychology Department, Université du Québec en Outaouais (UQO), Gatineau, QC J8X 3X7, Canada; 2 Interdisciplinary Health School, Université du Québec en Outaouais (UQO), 283 Boul. Alexandre-Taché, Gatineau, QC J8X 3X7, Canada; 3 Psychosocial Medicine Research Center, Centre Intégré de Santé et Services Sociaux de l’Outaouais (CISSSO), Gatineau, QC J8T 4J3, Canada; 4 Institut du Savoir de Montfort-Research Hospital, Ottawa, ON K1K 0T2, Canada; 5 Centre de Recherche de l’Institut Universitaire en Santé Mentale de Montréal IUSMM, Montreal, QC H1N 3V2, Canada; 6 Cyberpsychology Laboratory, Université du Québec en Outaouais (UQO), Gatineau, QC J8X 3X7, Canada

**Keywords:** virtual reality, virtual environment, validation, Social Physique Anxiety, obesity

## Abstract

State Social Physique Anxiety (SPA), in contrast to Trait SPA, is triggered by specific situations that elicit SPA. To date, no research has used virtual reality (VR) to recreate a situation that may elicit State SPA. The purpose of this study is to validate a virtual environment (VE) that simulates an anxiogenic situation to induce State SPA in women with obesity and high SPA. The high SPA group consisted of 25 self-identified women living with obesity and high Trait SPA. The low SPA group consisted of 20 self-identified women with low SPA. All participants were immersed in a virtual swimming pool environment for 10 min using a virtual reality headset. After the immersion, State SPA and fear of being negatively judged felt during immersion were measured with self-report questionnaires. A questionnaire assessing unwanted negative side effects was administered before and after the immersion. Using an ANCOVA with Trait SPA as covariate, State SPA was found to be significantly higher in the high SPA group. Fear of being judged negatively was also significantly higher in the high SPA group. Unwanted negative side effects scores did not increase post-immersion in either group. This study documents the validity of a novel VE for inducing State SPA in women with obesity and high SPA.

## 1. Introduction

Social Physique Anxiety (SPA) refers to the anxiety experienced when an individual feels observed or judged by others based on their physical appearance [1]. SPA can have many consequences including body image devaluation, low self-esteem, and social isolation. It is often associated with eating disorders and depression [2]. For people with obesity, high SPA is also associated with less time spent being physically active [3], less motivation to participate in weight loss programs, and lower exercise self-efficacy [4]. In Western societies, societal expectations of women’s physical appearance, as seen in the media, promote unrealistic thinness. Such social conditioning could significantly affect women living with obesity, most likely resulting in high SPA for individuals with obesity [5,6].

Given that cognitive behavior therapy (CBT) is a preferred intervention for the treatment of social anxiety [7] and that SPA is considered as a subtype of social anxiety [1], CBT may also be appropriate for SPA. CBT for social anxiety includes several psychotherapy techniques, with exposure being the most prominent [8]. The goal of exposure is to help patients confront and develop new and more appropriate mental representations of the feared stimuli [8]. Usually, exposure is conducted in vivo, meaning in real life settings, where the patient is gradually exposed to fearful social situations. For example, for SPA, common in vivo exposure scenarios include trying on a bathing suit versus a sweater [9]. In vivo exposure is plagued by problems such as being costly, time-consuming, and difficult to implement. In addition, in vivo exposure raises ethical issues about confidentiality, and people may be reluctant to participate in in vivo exposure [10]. To overcome these difficulties, researchers and clinicians have begun to use virtual reality (VR) as a tool to conduct virtual reality-based exposure, also referred to as in virtuo exposure [11].

In virtuo exposure allows for an ecological, systematic, gradual approach to a stimulus. A major advantage of VR is the ability to conduct exposure in situations that may be too difficult, too expensive, or too long to recreate in vivo. For instance, going to a pool where everyone else appears thin and pays attention to the individual. Although people immersed in VR are aware that the situation is generated by a computer, they feel, think, and react as if they were “in the real world” [12,13,14,15]. This phenomenon is related to the sense of presence, which can be defined as a perceptual illusion based on the illusion of plausibility (i.e., the feeling that what is happening in a virtual environment is actually happening) and the illusion of place (i.e., the feeling of actually being in the place recreated by the virtual environment). Both illusions are closely associated to the immersive nature of the experience, which refers to the technology’s capacity to replicate congruent multisensory stimuli and shield user’s attention from the objective laboratory setting.

VR has been used for SPA by Kroon et al. [16], and other studies used VR to induce, assess, and treat social anxiety [11,15,17,18]. However, VR has never been used to induce State SPA. As part of a broader research effort leading to another study not reported here (protocol available on ClinicalTrials.gov identifier: NCT04630184), a novel virtual environment (VE) was developed for in virtuo exposure that replicates a situation and stimuli that may induce SPA. The current study aimed to test whether the virtual environment could induce State SPA in women with obesity and SPA while not inducing negative side effects. The first hypothesis was that the use of a VE designed to induce SPA will result in higher State SPA scores, as measured by the validated State Social Physique Anxiety Scale [19], post-immersion on participants with high SPA (self-identified women with obesity and high SPA) compared to participants with low SPA (self-identified women without obesity and with low SPA). The second hypothesis was that an immersion in a VE designed to induce SPA will result in a higher fear of being negatively judged in the participants with high SPA compared to those with low SPA, as assessed with a question addressing fear of being negatively judged. The increase in unwanted negative side effects induced by the immersion in VR (commonly referred to as cybersickness) was also analyzed to document the safety of the VE.

## 2. Materials and Methods

### 2.1. Participants

Initially recruited for the other study, participants in the high SPA group were female self-identified; aged 18 to 45 years; had a body mass index (BMI) ≥ 30 kg/m^2^; engaged in ≤150 min of moderate-to-vigorous physical activity per week; and were able to attend a supervised exercise session twice a week at the Université du Québec en Outaouais (UQO, Gatineau, QC, Canada) and a psychological intervention for 12 consecutive weeks. Recruited specifically for the current study, participants in the low SPA group were female self-identified; between 18 and 45 years; spoke and understood French; and felt no or little discomfort when wearing a swimsuit. This last criterion was assessed with the following question, adapted from the Social Physique Anxiety Scale (SPAS) using a Likert scale from 1 (not at all) to 5 (extremely), “Please answer the following question: When wearing a bathing suit, I often feel nervous about the shape of my body”. The use of this last criterion helped to ensure that the low SPA group participants had no or little anxiety about the situation simulated by the VE, which was a swimming pool environment. The exclusion criteria were suffering from severe motion sickness, epilepsy, vestibular disorder, being pregnant, having intellectual disabilities, or severe and persistent psychiatric problem (psychosis, bipolar disorder).

A total of 25 women were recruited for the high SPA group and 20 women for the low SPA group. Recruitment was conducted through posters/advertisements on social media (e.g., Facebook, UQO intranet portal, Twitter), bulletin boards at the first author’s university, grocery stores, convenience stores, gas stations, and libraries. High SPA group participants were recruited over a period of 18 months (July 2021 to December 2022), and low SPA group participants were recruited over a period of 4 months (March to June 2022). The sample size for the current study was not set a priori based on a power analysis, but it provided sufficient power (at beta = 0.80) to detect large effect sizes (Cohen f = 0.40) at a significance level of 0.05 when comparing two groups (high SPA and low SPA).

### 2.2. Procedure

The current study was conducted at UQO and approved by the UQO Ethics Committee (project numbers: 2021-1591 and 2022-2058). The data of the high SPA group participants were collected during their first participation in the other study, and all participants consented to the secondary use of their data for the current study.

Participants in the high SPA and low SPA groups provided informed consent prior to the start of the study assessments. Prior to immersion, participants in both groups (high SPA and low SPA) completed questionnaires assessing sociodemographic data, body esteem, weight bias internalization, immersion predisposition, and Trait SPA. Their weights and heights were measured by a research assistant. As part of the pre-immersion roll-out of the other study, participants in the high SPA group performed 30 min of exercise on an ergocycle. Following the exercise and prior to the start of the immersion, participants completed a self-report questionnaire measuring pre-immersion symptoms of unwanted negative side effects of immersions in VR. Participants in the low SPA group also completed this pre-immersion self-report questionnaire but did not perform the exercise. For the VR immersion, participants of both groups physically sat on a chair in the laboratory room and were equipped with the VR headset. Before the immersion began, each participant was given the following instructions: “You are sitting in a comfortable chair. At the end of this instruction, if you are still comfortable, you will be immersed in VR with the headset covering your eyes. You are wearing a bathing suit and sitting on a pool deck next to a swimming pool. You will be observed by various virtual characters for 10 min. If you feel uncomfortable with the situation, we can stop at any time”. They were told to remain seated on the chair during the immersion, but they could move their body and look around.

At the end of the VR immersion, participants in both groups completed questionnaires to assess their State SPA, fear of being judged negatively, and unwanted negative side effects.

### 2.3. Virtual Environment

**Figure 1:**
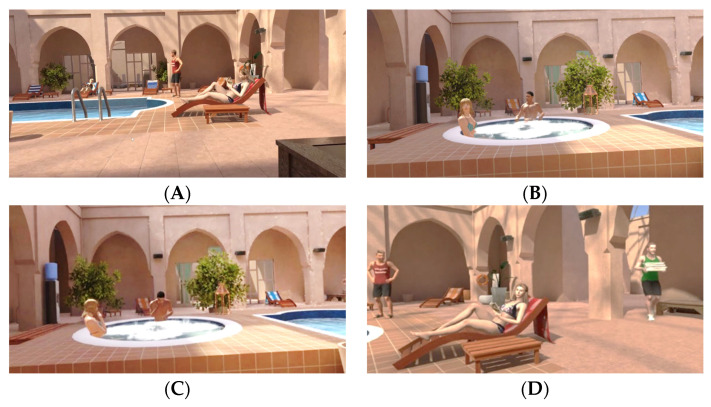
Virtual environment as seen by the participant. (**A**) Central view of the VE, (**B**) Left side of the VE, (**C**) Couple laughing, and (**D**) Right side of the VE.

To reduce the novelty effect induced by participants’ first uses of VR technology, each participant was immersed in the VE for one minute before the scenario began. The scenario then lasted 10 min and depicted a situation often described as anxiogenic [20]. The design of this VE consisted of a virtual scene depicting a swimming pool in a luxurious building. Participants were immersed as if they were sitting on a chair next to the pool. They were observed by several virtual characters. A lifeguard standing on the right side of the pool looked at them from time to time. There were a few deck chairs around the pool, and two of them were occupied. One was occupied by a woman lying on her back reading a book and occasionally looking at the participant. The second deck chair was occupied by another woman who was sleeping (Figure 1A). To the left of the participant was a couple in a hot tub (Figure 1B). Spa background music was audible in addition to birds and hot tub sound effects. Participants could not see their virtual bodies, but they could see their virtual chair. The scenario was standardized and began with the lifeguard crossing/uncrossing his arms frequently, looking at the participant twice, then looking at the hot tub, and then looking at the woman lying in a deck chair in front of him. After 1 min, the couple in the hot tub began talking to each other. The participants could hear their voices, but their discussion was unintelligible. At 2 min, the man in the hot tub began to stare at the participant for a few seconds. Then, at 3 min of immersion, the woman reading a book looked at the participant for a few seconds and then returned to her reading. At 4 min, the couple began to laugh together several times (Figure 1C). The couple looked at the participant from time to time. They continued to laugh and stare at the participant for another 2 min. Then, at 6 min, a male hotel employee entered from the right side of the virtual environment (Figure 1D), brought towels, and left them on a coffee table (Figure 1A). As he left the towels, he stared at the participant. Then, the hotel employee left the pool deck, and the lifeguard started looking at the participant from time to time, as did the couple sitting in the hot tub. At 9 min, the woman who was reading picked up her cell phone and started using it. Finally, at 10 min, the participant reached the end of the immersion.

It was not possible for the participant to interact verbally or physically with the virtual characters. The VE was displayed using an Oculus Rift 2 headset (Oculus, Irvine, CA, USA) connected to a computer Intel^®^ Core^(TM)^ i7-10700 with a 2.9 GHz core processor unit and 32.0 Gb of RAM and an NVIDIQ GeForce GTX 1660 SUPER graphics card. The lab room had only a desk, a chair for the research assistant, and an armless chair for the participant.

### 2.4. Measures

#### 2.4.1. Dependent Variables: State SPA Level and Measures of the Virtual Experience

The validated State Social Physique Anxiety Scale [19] was administered. Using a Likert scale ranging from 1 (not at all) to 5 (extremely), all participants had to rate how each of the nine items applied to them. High scores on the State SPA scale indicated a greater presence of SPA induced by the situation. In this study, Cronbach’s alpha was 0.942.

To confirm that the VE elicited State SPA, participants were also asked to rate the fear of being negatively judged by others that they experienced during the immersion. After immersion, all participants answered the following question using a 5-point scale ranging from 1 (not at all) to 5 (extremely) “Please indicate the extent to which your immersion in the VE resulted in a fear of being negatively judged by others because of your body type”.

Unwanted negative side effects induced by the immersion in VR (i.e., cybersickness) were measured using the validated 16-item Simulator Sickness Questionnaire (SSQ) [21,22]. In this study, the Cronbach’s alpha calculated from the pre-immersion scores was 0.828, and the Cronbach’s alpha calculated from the post-immersion scores was 0.71. This questionnaire was scored using the approach recommended by Bouchard et al. (2021) [23], which includes scoring a nausea factor, an oculomotor factor, and a total score. Responses were given on a Likert scale ranging from 0 (not at all) to 3 (extremely). Raw scores for each factor were analyzed separately. A high score within each factor was associated with the presence of an unwanted negative side effect, commonly referred to as cybersickness. Any interruption of the VR immersion from the participant was to be recorded by the research assistant, as well as the reason for the interruption.

#### 2.4.2. Covariates: Sociodemographic and Clinical Measures

A sociodemographic questionnaire was administered to collect participants’ age, educational level, marital and employment status, and income. Anthropometric data were collected by a research assistant. Each participant’s height and weight were measured using a measuring rod and a Tanita electronic scale (light clothing and no shoes). These data were used to calculate body mass index (BMI = weight (kg)/height (m)^2^).

Trait SPA was assessed using the validated 9-item Trait Social Physique Anxiety Scale (SPAS) [24]. Participants were asked to rate how applicable each item was to them using a Likert scale ranging from 1 (not at all) to 5 (extremely). High scores on the Trait SPA scale indicated a greater presence of SPA as a Trait. (Cronbach’s alpha calculated based on results of this study was 0.933.)

The validated 11-item Weight Internalization Bias Scale (WIBS) [25] was used to assess participants’ internalized weight bias. Participants were asked to rate how applicable each item was to them, using a Likert scale ranging from 1 (strongly disagree) to 7 (strongly agree). High scores on the WIBS indicated a greater presence of internalized weight bias. In this study, Cronbach’s alpha was 0.941.

The validated 10-item Body Appreciation Scale (BAS) [26] was used to assess participants’ body image. Participants were asked to rate how applicable each item was to them using a Likert scale ranging from 1 (never) to 5 (always). High scores on the validated BAS indicated greater body esteem. In this study, Cronbach’s alpha was 0.957.

Immersion tendencies were assessed using a validated 18-item questionnaire assessing attentional focus, involvement, emotions, and tendency to play video games [27]. Participants were asked to rate how applicable each item was to them using a Likert scale ranging from 1 (never) to 7 (often), which was then summarized into 4 factors: focus, involvement, emotions, and gaming. High scores on the Immersion Tendencies Questionnaire were associated with an increased sense of presence. In this study, Cronbach’s alpha was 0.851.

To assess the sense of presence in VR, participants were asked to complete the validated Presence Questionnaire ITC-SOPI [28]. This is a 44-item questionnaire with a five-point Likert scale (1: strongly disagree—5: strongly agree) assessing spatial presence, engagement, naturalness, and negative effects. Spatial presence, engagement, and naturalness scores close to 5 (which is the maximum score) indicated a high sense of presence, and a score close to 0 for negative effects was expected to characterize a safe environment. Item 36 was removed from the analysis because participants were not able to move an object, and it was judged to be inapplicable for this study. In this study, Cronbach’s alpha was 0.922.

### 2.5. Statistical Analyses

IBM SPSS v28 was used to perform statistical analysis. Results were considered statistically significant if *p* < 0.05. The z-scores of kurtosis and skewness of the continuous variables were analyzed to verify the normality of their distribution [29]. Cronbach’s alpha was calculated for all validated questionnaires. Cronbach’s alpha was defined as low (≤0.59), marginal (0.60–0.69), acceptable (0.70–0.79), good (0.80–0.89), and excellent (≥0.90) [30]. The Chi-squared test of independence was used for categorical variables to assess differences between groups. The assumption of homogeneity of variance was tested using Levene’s test for equality of variances; then, the independent samples *t*-test was used for numerical variables to test for differences between groups. Effect sizes were estimated using Cohen’s d. A d-value of 0.2 was considered a small effect size and 0.8 was considered a large effect size [31]. Repeated measures ANOVAs were used to compare pre- and post-immersion cybersickness within and between groups. Partial eta-squared (η_p_^2^) was calculated as an estimate of effect size, with η_p_^2^ = 0.01 indicating a small effect and η_p_^2^ = 0.14 indicating a large effect [31,32]. Sensitivity analyses were also conducted with age and BMI as covariates, and the results did not change. To control for pre-experiment differences in participants’ SPA, post-immersion State SPA was analyzed using an ANCOVA with Trait SPA as a covariate.

## 3. Results

### 3.1. Sample Characteristics

Sociodemographic and clinical data describing the sample are shown in Table 1. Participants of the high SPA group had significantly higher BMI, were older, and scored higher on Trait SPA and the WBIS and lower on the BAS than participants in the low SPA group. Note that the WBIS and BAS correlated with Trait SPA at 0.89, and hence were not used as covariates. Regarding predisposition to immersion, high SPA participants scored lower than low SPA participants on the factors of focus and involvement. No significant differences between groups were found for emotions and game factors (Table 1). The sense of presence induced by the environment was not significantly different between the two groups. One participant in the high SPA group asked to stop the immersion after a period of 6 min, mentioning that she was too socially uncomfortable with the situation.

### 3.2. Dependent Variables

Controlling for Trait SPA, the ANCOVA revealed significantly higher State SPA scores for the high SPA group than the low SPA group (Table 2). The high SPA group also scored significantly higher on the anxiety question than the low SPA group.

Scores on the SSQ did not increase significantly post-immersion in either group (Table 3). Higher scores were found for all factors in the high SPA group compared to the low SPA group, with significant time and conditions main effects and interaction effects for the nausea and total cybersickness factors (Table 3). Scores on the SSQ post-immersion did not differ according to the conditions (nausea *t*_(42.63)_ = 1.46 *p* = 0.08, oculomotor *t_(_*_43)_ = −0.01 *p* = 0.50, and total *t*_(43)_ = 0.44 *p* = 0.33).

## 4. Discussion

This study was carried out to validate whether the VE designed for clinical interventions can induce State SPA in women with obesity and SPA. After controlling for Trait SPA, State SPA was found to be significantly higher for the high SPA group, which provides preliminary evidence that the environment is capable of inducing State SPA in women with obesity and SPA. In addition, the fear of being judged negatively by others based on physical appearance was also higher for the high SPA group. Being in a VR pool deck and being observed by virtual people have the ability to induce SPA in women with obesity and high Trait SPA compared to women with low Trait SPA. The hypotheses that State SPA and fear of being judged negatively would be higher in the high SPA group than in the low SPA group following an immersion in VE were thus confirmed. This suggests that a VE is likely to induce higher State SPA in individuals who are more prone to experiencing SPA and preoccupied with their bodies than in individuals who are less prone to such preoccupations.

Regarding unwanted negative side effects and safety of immersions in the VE, results found a decrease in negative side effects (commonly referred to as cybersickness) for the high SPA group. Higher baseline scores for symptoms of nausea and the total score of the SSQ were found in the high SPA group, which can be explained by the 30 min of physical activity performed prior to immersion as part of the roll-out of the other study. Exercise can increase symptoms measured by the SSQ [21]. These higher scores could also be supported by participants in the high SPA group feeling slightly anxious before the immersion began, knowing that they would be immersed in a situation they might be afraid of [21]. However, all scores of the high SPA group decreased during immersion in the VE and finally reported equivalent scores post-immersion compared to the low SPA group, thus demonstrating that it can be used safely for women with obesity and high Trait SPA.

Looking at the presence scores in both groups, the results showed that the participants were equally engaged in the VE. With scores above 3/5 for spatial presence, engagement, and naturalness factors, all participants showed satisfactory results as equivalent compared to published satisfactory results [33]. The scores for negative effects presence confirmed what was observed with the SSQ that the VE was a safe environment. Overall, it can be concluded that immersion in the VE can create an experience of good quality.

This new VE has potential for use in clinical applications. For example, it could be used for exposure-based CBT by allowing psychotherapists to work with individuals who have obesity to overcome social fears and engage in physical exercise more actively. Additionally, it may be helpful for other populations facing SPA, including individuals with body image disturbances (e.g., anorexia), body dysmorphic disorder, visible scars or physical disabilities, as well as social anxiety disorder. Clinical researchers can also utilize this VE to manipulate SPA experimentally, replicating or refining Koon et al.’s [16] study, or in conjunction with brain imaging techniques. To strengthen the VE, some improvements can still be made. For example, it would be relevant to add the possibility for participants to virtually see their body, or at least a part of it. Indeed, previous research has shown that the inclusion of virtual hands and feet helps to achieve a stronger sense of embodiment, which is a factor known to contribute to the sense of presence [34]. Another way to increase the quality of the immersive experience could be to allow the participant to walk around the environment and/or to pick up and manipulate objects left around, such as towels dropped on the coffee table, or to socially interact with virtual characters [34].

## 5. Limitations

The main limitation of this study is that State SPA was not measured pre-immersion in the low SPA group. For this reason, Trait SPA was used as a covariate to estimate pre-immersion State SPA. Although assessing State SPA pre-immersion in all participants would have been ideal, it could have introduced bias into the post-immersion responses. Participants in the high SPA were subjected to physical exercise before the immersion, as required by their participation in another study, which was not the case for low SPA participants. This is a methodological difference between the two conditions and its impact observed on the baseline scores of unwanted negative side effects was discussed earlier. If the study was to be repeated, a better approach for conducting the experiment would be to keep apart the physical activity intervention from the VR session for all participants. Another limitation concerns sample selection. More specifically, participants were not randomized between the two groups, and thus, these groups were different prior to immersion. Participants in the high SPA group had to meet more selection criteria (e.g., being obese and having a high Trait SPA) than those in the low SPA group. These differences in inclusion criteria may have contributed to the significant differences in SPA and fear of being judged that were found following the VE immersions. Replicating the study but matching participants on as many variables as possible (e.g., age, socio-economic status, life stressors, lifestyle and physical activity, immersive tendencies, cybersickness) may allow to better isolate the impact of the immersion on people with high and low SPA. In addition, this study is limited by the lack of physiological measures (e.g., heart rate, cortisol) and reliance on self-report measures to assess anxiety. Documenting physiological differences in anxiety responses would add strength to what was found based on self-report measures. Given that the results of the present study indicate that this novel VE induces a higher emotional response in individuals who have a higher SPA, future studies should explore additional differences in individuals with high and low Trait SPA. We found differences in age and BMI, but sensitivity analyses showed it did not influence the conclusions. Also, the participants were only self-identified females, making it impossible to know how males might respond to such a VE. To improve generalization of the results, this study should be replicated with a larger sample size and include men. Additionally, ethnicity could have been assessed as part of the sociodemographic data to obtain a better picture of the participants, knowing that SPA is not homogeneous across different ethnicities [35].

## 6. Conclusions

In summary, with higher State SPA scores and higher fear question scores for the high SPA group than for the low SPA group, these results indicate that a novel virtual pool scenario can induce State SPA in larger-bodied women with high SPA. In addition, the VE induced little or no cybersickness. Overall, the results showed that the VE safely induced anxiety related to physical appearance and thus could be used in further studies investigating the psychotherapeutic effects of in virtuo exposure to SPA in women with obesity. This VE could also be used for in virtuo CBT exposure.

## Figures and Tables

**Table 1:** High SPA and low SPA groups’ descriptive information (N = 45).

			Statistics		
Measures	High SPA Group (*n* = 25)	Low SPA Group (*n* = 20)	χ^2^	*t_(43)_*	*d*
Married *n* (%)	13 (52.0)	3 (15)	14.37 **	-	-
University degree *n* (%)	15 (60.0)	20 (100)	10.29 **	-	-
Full time job *n* (%)	24 (96.0)	6 (30.0)	25.56 ***	-	-
Income ≥CAD 100,000 *n* (%)	14 (56.0)	5 (25.0)	19.43 **	-	-
BMI Mean (s.d.)	39.82 (6.19)	22.08 (2.42)	-	13.01 ***	4.89
Age Mean (s.d.)	40.56 (4.60)	26.15 (6.05)	-	9.07 ***	5.29
Trait SPA Mean (s.d.)	34.92 (4.31)	19.30 (4.89)	-	11.38 ***	4.57
WBIS Mean (s.d.)	4.61 (0.82)	1.72 (0.59)	-	13.76 ***	0.73
BAS Mean (s.d.)	2.53 (0.51)	3.97 (0.51)	-	−9.41 ***	0.51
Immersive Tendencies					
Focus Mean (s.d.)	19.92 (5.42)	25.10 (3.35)	-	−3.93 ***	4.62
Involvment Mean (s.d.)	13.44 (5.28)	20.50 (5.51)	-	−4.37 ***	5.39
Emotions Mean (s.d.)	14.48 (4.40)	16.90 (4.70)	-	−1.78	4.53
Gaming Mean (s.d.)	6.08 (2.97)	7.42 (2.82)	-	−1.52	2.90
Presence			***t***	***df***	***d***
Spatial Presence Mean (s.d.)	2.97 (0.93)	3.36 (0.50)	−1.43	43	0.50
Engagement Mean (s.d.)	3.31 (0.54)	3.44 (0.36)	−0.99	43	0.26
Naturalness Mean (s.d.)	3.03 (0.67)	3.49 (0.68)	−2.28	43	0.68
Negatives Effects Mean (s.d.)	1.71 (0.50)	1.70 (0.63)	0.09	43	0.03

High SPA group = participants with obesity and high State Social Physique Anxiety. Low SPA group = participants with low Social Physique Anxiety. BMI = Body Mass Index. WBIS = Weight Internalization Bias Scale. BAS = Body Appreciation Scale. n = sample size. Data of *t*-test are expressed as t_(*df).*_ *df:* degrees of freedom. *d:* Cohen’s d. *s.d.:* standard deviation. ** *p* < 0.01. *** *p* < 0.001.

**Table 2:** High SPA and low SPA group comparisons of SPA levels and virtual experiences measured after immersion.

Measures	High SPA Group (*n* = 25)	Low SPA Group (*n* = 20)	*F_(1,43)_*	*p*	*η_p_^2^*
State SPA Mean (s.d.)	34.84 (5.31)	17.20 (4.36)	6.34	0.016	0.13
			***t_(42)_***	***p***	***d***
Fear Question Mean (s.d.)	2.96 (0.96)	1.55 (0.51)	5.92	<0.001	1.79

*n* = sample size. *s.d.* = standard deviation. *ηp*^2^ = Partial eta-squared. Data of ANCOVA are expressed as *F_(df between groups, df within groups)_*. Data of *t*-test are expressed as *t_(df)_*. *df*: degrees of freedom. *d*: Cohen’s d.

**Table 3:** Raw score of unwanted negative side effects pre- and post-virtual immersion for women in the high SPA and low SPA groups.

Measures	High SPA Group (*n* = 25)		Low SPA Group (*n* = 20)		*F_(1,43)_*	*p*	*η_p_^2^*
	Pre	Post	Pre	Post			
Nausea Mean *(s.d.)*	3.12(2.24)	0.64(0.91)	0.30(0.47)	0.30(0.66)			
Main effect of time					24.04	<0.001	0.36
Main effect of conditions					26.02	<0.001	0.38
Conditions x time					24.04	<0.001	0.36
Oculomotor Mean *(s.d.)*	3.28(2.54)	2.84(2.51)	2.40(2.06)	2.85(2.25)			
Main effect of time					0.001	0.98	0.00
Main effect of conditions					0.41	0.52	0.01
Conditions x time					4.15	0.05	0.09
Total Mean (s.d.)	6.40(4.15)	3.48(2.58)	2.70(2.22)	3.15(2.43)			
Main effect of time					12.52	<0.001	0.23
Main effect of conditions					5.84	0.02	0.12
Conditions × time					23.31	<0.001	0.35

*n* = sample size. s.d. = standard deviation. *η_p_^2^ =* Partial eta-squared. Data of repeated ANOVA are expressed as *F_(df between groups, df within groups)._ df:* degrees of freedom.

## Data Availability

The data that support the findings of this study are available upon request addressed directly to the relevant Research Ethics Board (comite.ethique@uqo.ca). The dataset is not publicly available due to privacy and ethical restrictions.
